# Systematic Review of Extracorporeal Membrane Oxygenation in Adult Sickle Cell Disease

**DOI:** 10.3390/jcm14196725

**Published:** 2025-09-24

**Authors:** Safa Khalil Ebrahim Al Taitoon, Kannan Sridharan

**Affiliations:** 1 Adult Critical Care Medicine, Salmaniya Medical Complex, Manama, Bahrain; 2 Department of Pharmacology & Therapeutics, College of Medicine & Health Sciences, Arabian Gulf University, Manama P.O. Box 26671, Bahrain; skannandr@gmail.com

**Keywords:** extracorporeal membrane oxygenation, sickle cell disease, acute chest syndrome, pulmonary hypertension, refractory respiratory failure

## Abstract

**Background:** Sickle cell disease (SCD) is a hereditary hemoglobinopathy associated with life-threatening complications such as acute chest syndrome (ACS), which may necessitate extracorporeal membrane oxygenation (ECMO) in refractory cases. Despite growing use, ECMO in SCD remains challenging due to risks of hemolysis, thrombosis, and anticoagulation complications. This systematic review consolidates existing evidence on ECMO outcomes in SCD, focusing on indications, complications, and survival. **Methods:** A systematic search of MEDLINE, Cochrane CENTRAL, and Google Scholar was conducted up to January 2025, identifying case reports/series on ECMO use in SCD. Studies reporting venovenous (VV) or venoarterial (VA) ECMO for acute cardiopulmonary failure were included. Data on demographics, laboratory findings, management, and outcomes were extracted. Quality assessment was performed using the Joanna Briggs Institute checklist. **Results:** Sixteen case reports (23 patients) were included. Most patients were female (65.2%), with ACS (47.8%) and pulmonary embolism (13.0%) as common ECMO indications. VV-ECMO (69.6% of cases) was primarily used for respiratory failure, with a 69% survival rate, while VA-ECMO (30.4%) had a 29% survival rate, often due to cardiogenic shock or cardiac arrest. Complications included hemorrhage (26.1%), neurological injury (21.7%), and thrombosis (13.0%). Exchange transfusion was frequently employed (43.5%), with post-ECMO echocardiography showing improved right ventricular function in survivors. **Conclusions:** VV-ECMO demonstrates favorable outcomes in SCD-related respiratory failure, whereas VA-ECMO carries higher mortality risks. Careful patient selection, anticoagulation management, and multidisciplinary coordination are essential. Larger prospective studies are needed to refine ECMO utilization in this high-risk population.

## 1. Introduction

Sickle cell disease (SCD) is a hereditary hematologic disorder caused by a mutation in the β-globin gene, resulting in the production of sickle hemoglobin (HbS). Under conditions of low oxygen tension, HbS polymerizes, leading to the sickling of red blood cells (RBCs), causing cellular rigidity, hemolysis, and microvascular occlusion. This process promotes chronic inflammation, endothelial dysfunction, and hypercoagulability, ultimately leading to organ damage, including cardiopulmonary complications such as pulmonary hypertension (PH) and right ventricular (RV) dysfunction [1,2]. Globally, SCD affects millions of individuals, with the highest prevalence in sub-Saharan Africa, India, and the Middle East, and is increasingly recognized as a major contributor to premature morbidity and mortality in high-income countries as well, owing to improved survival into adulthood. Cardiopulmonary complications remain a leading cause of death in adults with SCD, underscoring the clinical need for advanced rescue strategies when conventional therapy fails.

Acute chest syndrome (ACS), one of the most common and life-threatening complications in SCD, is associated with respiratory failure, requiring advanced life support such as mechanical ventilation. ACS often results from pulmonary vaso-occlusion, infection, or fat embolism, all of which contribute to hypoxemia and increased pulmonary vascular resistance (PVR) [3,4]. Chronic hemolysis further depletes nitric oxide, exacerbating vasoconstriction and endothelial dysfunction, which can precipitate PH and RV strain [1,5]. The resulting circulatory strain, combined with severe anemia, leads to a hyperdynamic state that can further stress the right heart, contributing to maladaptive remodeling over time [6,7]. Standard management approaches for ACS and related cardiopulmonary complications include oxygen supplementation, antibiotics, pain control, and blood transfusion or exchange transfusion. However, despite aggressive supportive therapy, a subset of patients progress to refractory hypoxemia or circulatory collapse, highlighting the need for salvage interventions such as ECMO.

Extracorporeal membrane oxygenation (ECMO) is an advanced life support modality that has been increasingly utilized in the management of SCD patients with refractory respiratory failure, particularly those with severe ACS or PH. While ECMO is commonly used for acute respiratory and circulatory failure in conditions such as pulmonary embolism (PE) and severe PH, its use in SCD has been limited by concerns about hemolysis, thrombotic complications, and challenges in anticoagulation management [8,9]. The interplay of chronic hemolysis, coagulation abnormalities, and endothelial dysfunction creates a particularly complex milieu during ECMO support. Hemolysis may be amplified by shear stress from the extracorporeal circuit, further increasing free hemoglobin and nitric oxide scavenging. Endothelial dysfunction enhances vasoconstriction and inflammatory responses, while the hypercoagulable state heightens the risk of thrombosis, even in the presence of systemic anticoagulation. These overlapping mechanisms complicate circuit durability, anticoagulation protocols, and overall outcomes, distinguishing ECMO in SCD from other critical care populations. Furthermore, decision-making around ECMO initiation in SCD is complicated by ethical considerations, including resource allocation, the presence of multi-organ dysfunction, and uncertainty about long-term prognosis in patients with advanced disease. These unique challenges underscore the importance of systematically reviewing the evidence to guide practice. However, emerging case reports and retrospective studies have highlighted ECMO as a potentially life-saving intervention for SCD patients experiencing refractory respiratory failure [10].

Data from the Extracorporeal Life Support Organization (ELSO) registry suggest that survival outcomes for SCD patients on ECMO are comparable to those for non-SCD patients with similar indications [11,12,13]. Nevertheless, challenges remain in the application of ECMO in SCD patients, including the high incidence of circuit-related complications, difficulties in maintaining optimal anticoagulation balance, and ethical dilemmas regarding ECMO eligibility for patients with advanced organ dysfunction. Importantly, no prior synthesis systematically compared survival differences between veno-venous (VV) and veno-arterial (VA) ECMO in SCD. Importantly, despite increasing case-level evidence, no prior synthesis has systematically evaluated survival differences between veno-venous (VV) and veno-arterial (VA) ECMO in this population, leaving clinicians uncertain about which modality may confer the greatest benefit. Highlighting this gap, our review specifically evaluates pooled outcomes using an ECMO modality, thereby providing novel insights into which patients may derive the greatest benefit from this high-risk intervention.

This systematic review aims to consolidate the current literature on the use of ECMO in SCD patients, focusing on its indications, clinical outcomes, complications, and management strategies. By synthesizing available evidence, the review seeks to improve the understanding of ECMO’s role in SCD management and contribute to the development of future clinical guidelines and research. In doing so, it not only addresses an urgent gap in critical care literature but also provides a framework for future multicenter collaborations and the eventual integration of ECMO into disease-specific guidelines for SCD. In addition to homozygous SCD, some patients may present with compound heterozygous states such as sickle cell–beta-thalassemia, which share pathophysiologic features of vaso-occlusion, hemolysis, and cardiopulmonary complications, though with variable clinical severity. A few of the case reports included in this review described ECMO use in such SCD/beta-thalassemia patients, and these have been analyzed together, given their overlapping clinical manifestations in the context of refractory respiratory failure.

## 2. Methods

### 2.1. Study Design and Ethics

The protocol for this review was registered in the Open Science Framework (registration ID: WRPX4) [link: https://osf.io/j3mxn (accessed date: 3 January 2025)] [14]. A systematic search for case reports or series was conducted using the following electronic databases: Medline, Cochrane CENTRAL and Google Scholar with the search strategy: (ECMO [Title/Abstract] OR extracorporeal membrane oxygenation [Title/Abstract]) AND (pulmonary hypertension [Title/Abstract] OR sickle cell disease [Title/Abstract]). The latest search was carried out on 10 January 2025. No restrictions were placed on either language or year of publication. Case reports/series reporting the use of ECMO (any type) in SCD patients were included.

### 2.2. Study Procedure

Two authors independently obtained the following details from each of the case reports/series. Any discrepancies were resolved through discussion. The following details were obtained from the case reports: demographic characteristics, laboratory investigations, and management strategies for each patient. Demographic details included age, gender, and country of origin, along with documented comorbidities and initial diagnoses. Laboratory investigations encompassed hemoglobin levels, platelet counts, markers of hemolysis such as lactate dehydrogenase, acid-base status including lactate and bicarbonate, troponin levels, and percentage of hemoglobin S (HbS%). Additional laboratory parameters included renal and hepatic function tests, inflammatory markers, coagulation profiles, and microbiological studies when available. Management strategies were systematically recorded, including initial treatments such as antibiotics, analgesia, hydration, blood transfusions, and exchange transfusions. ECMO utilization was documented with specifications on type (VV or VA), anticoagulation protocols, duration of support, and complications encountered during therapy. Pre- and post-ECMO echocardiographic findings, clinical outcomes, and length of stay in intensive care and hospital settings were also extracted from each case report. This standardized data collection allowed for systematic evaluation of patient characteristics, disease severity, therapeutic interventions, and clinical outcomes across all cases. The Joanna Briggs Institute (JBI) critical appraisal checklist was used for assessing the quality of case reports included in this study [15]. This tool evaluates methodological quality across eight key domains: (i) clarity in describing patient demographic characteristics; (ii) adequacy of patient history and clinical presentation; (iii) clarity in the description of diagnostic methods and results; (iv) appropriateness and completeness of interventions or treatments; (v) detail in reporting clinical outcomes and follow-up; (vi) identification of adverse events or unanticipated outcomes; (vii) provision of clear takeaway lessons; and (viii) overall completeness and transparency of reporting. Each domain was assessed as “yes,” “no,” “unclear,” or “not applicable,” allowing for a structured and transparent evaluation of reporting quality. Discrepancies between reviewers were resolved through discussion and consensus.

## 3. Results

### 3.1. Search Results and Demographic Characteristics of Included Case Reports

**Figure 1:**
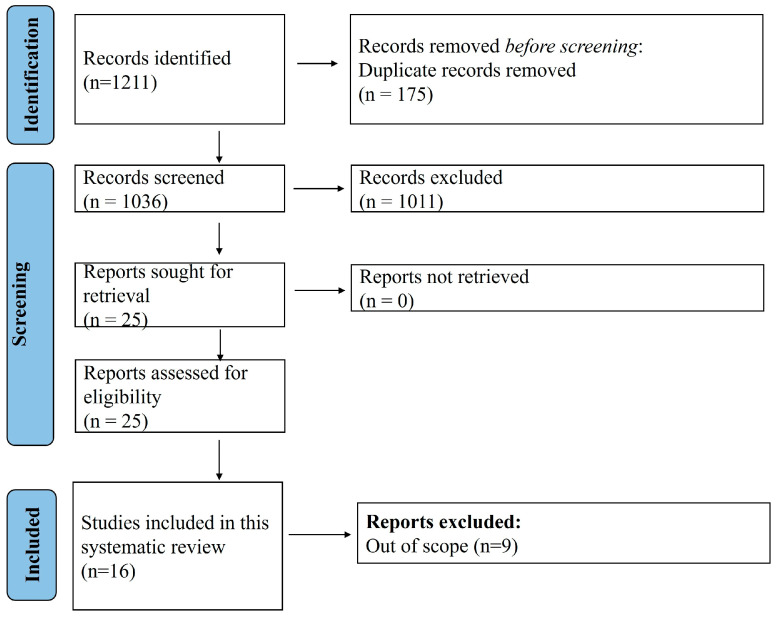
PRISMA flow diagram.

A total of 1211 articles were obtained with the search strategy, of which, finally, 16 case reports/series [16,17,18,19,20,21,22,23,24,25,26,27,28,29,30,31] were eligible for inclusion in this study (Figure 1). Table 1 summarizes 23 cases from 16 reports, detailing patient demographics, concomitant diseases, and initial diagnoses. Reports included patients from the USA, France, Germany, the UK, and Israel, with ages ranging from 18 to 60 years. Most patients were female (15/23, 65.2%), and several had underlying conditions such as beta-thalassemia (n = 4), chronic CKD (n = 2), rheumatic heart disease (n = 2), and iron overload (n = 2). Common initial diagnoses included VOC (n = 9), ACS (n = 6), pulmonary embolism (n = 3), and infections such as community-acquired pneumonia (n = 2) and urinary tract infections (n = 2). Conditions like cardiogenic shock (n = 1), fulminant colitis (n = 1), and acute respiratory failure (n = 2) were also noted. The table highlights the diverse clinical presentations and comorbidities in these cases, emphasizing the complexity of managing such patients. The JBI checklist assessment revealed that all except five cases included had high quality, meeting all the domains listed in the checklist.

A total of 16 reports were included in this study out of 1211 that were obtained with the search strategy.

### 3.2. Laboratory Investigation Findings in the Reported Cases

Table 2 summarizes the findings of the laboratory investigations including imaging modalities. The echocardiographic findings revealed signs of right ventricular strain including right ventricular dilation (n = 8), right ventricular dysfunction (n = 6), elevated pulmonary artery systolic pressure (n = 5), left ventricular dysfunction (n = 2) and elevated troponin (n = 1). Acute cor pulmonale was noted in 2 patients, while tricuspid regurgitation was reported in 4 patients.

CT chest findings predominantly showed bilateral pulmonary consolidations (n = 12), ground-glass opacities (n = 3), and pulmonary embolism (n = 3).

HbS% varied widely, with >70% in 3 patients and intermediate levels (30–70%) in 5 patients.

Other notable laboratory abnormalities included anemia (hemoglobin <8 g/dL) in 10 patients, thrombocytopenia in 4, elevated lactate dehydrogenase (LDH) in 6, and lactic acidosis in 3. Leukocytosis was observed in 7 patients, underscoring the underlying hematologic disturbances in this cohort. Additionally, troponin elevation was reported in 4 patients, potentially indicative of right ventricular strain.

### 3.3. Management and Outcomes Reported with ECMO

The management strategies and outcomes of 23 patients treated with ECMO in the case reports are outlined in Table 3. Initial treatment commonly included antibiotics (n = 14), hydration (n = 13), opioids (n = 13), intubation (n = 14), and exchange transfusion (n = 10). The primary indications for ECMO were ACS (n = 11), acute respiratory distress syndrome (n = 5), pulmonary embolism with shock (n = 4), and acute respiratory failure (n = 3).

VA-ECMO was used in 7 patients, primarily for cardiogenic shock secondary to PE (n = 4), while VV-ECMO was employed in 16 patients, mostly for ACS or acute respiratory distress syndrome (n = 15). Anticoagulation was administered in 10 patients, with no anticoagulation in 4 cases, and unclear status in 9.

Post-ECMO echocardiography showed improved RV function (n = 3), regression of acute cor pulmonale (n = 1), and 18 cases lacked post-ECMO echo data. Complications were frequent, including hemorrhage (n = 6), coagulopathy (n = 3), acute kidney injury (n = 3), neurological injury (n = 5), infections (n = 3), and thrombotic events (n = 3). Overall survival was 59% (n = 13/22, excluding Case 23 with incomplete data), with higher survival in VV-ECMO (n = 11/16, 68.75%) compared to VA-ECMO (n = 2/7, 28.6%). Length of stay (LOS) varied widely, with ICU stays ranging from 3 to >48 days and hospital LOS up to 44 days. Key additional findings included fat embolism (n = 3), cerebral fat embolism syndrome (n = 2), and allogeneic stem cell transplantation (n = 1).

In summary, VV-ECMO was the predominant modality (n = 16), primarily for ACS/ARDS, with a 69% survival rate (11/16). VA-ECMO (n = 7), used for cardiogenic shock/PE, had a 29% survival rate (2/7). Hemorrhagic and neurological complications were major contributors to mortality, particularly in VA-ECMO cases. The data underscore VV-ECMO’s favorable outcomes in respiratory failure, while VA-ECMO carried higher risks and poorer survival, likely reflecting the severity of underlying cardiac or thromboembolic pathology.

## 4. Discussion

### 4.1. Key Findings

Extracorporeal membrane oxygenation serves as a critical life-support modality for adult patients with SCD experiencing severe cardiopulmonary failure. This systematic review highlights key distinctions between VA and VV ECMO in terms of indications, outcomes, and complications, emphasizing the importance of tailored therapeutic strategies in this high-risk population. A novel and central finding of this review is the clear divergence in survival outcomes between ECMO modalities: pooled survival was substantially higher for VV ECMO (69%) compared to VA ECMO (29%). We included 23 case reports with detailed demographic, laboratory, and management data. Demographics revealed a predominance of female patients (n = 15), with ages ranging from 18 to 60 years, and cases originating from the USA (n = 10), Germany (n = 5), France (n = 4), the UK (n = 2), and Israel (n = 1). Common comorbidities included beta-thalassemia (n = 4), chronic kidney disease (CKD) (n = 2), and rheumatic heart disease (n = 2). Laboratory investigations frequently demonstrated anemia (Hb < 8 g/dL, n = 10), thrombocytopenia (n = 4), elevated lactate dehydrogenase (LDH, n = 6), lactic acidosis (n = 3), and troponin elevation (n = 4), with HbS% varying widely (30–90%). Initial diagnoses were primarily VOC (n = 9), ACS (n = 6), and PE (n = 3). Management strategies included exchange transfusion (n = 10), antibiotics (n = 14), hydration (n = 13), and ECMO support (n = 23), with VV-ECMO (n = 16) used mostly for ACS/ARDS and VA-ECMO (n = 7) for cardiogenic shock/PE. Complications such as hemorrhage (n = 6), neurological injury (n = 5), and infections (n = 3) were common, influencing outcomes, which showed an overall survival rate of 59%. Echocardiographic and CT findings frequently noted right ventricular dysfunction (n = 6) and bilateral pulmonary consolidations (n = 12), further guiding therapeutic decisions. This comprehensive data synthesis highlights the heterogeneity in presentation, laboratory derangements, and management challenges in this critically ill cohort.

### 4.2. Comparison with Existing Literature

Extracorporeal membrane oxygenation is increasingly being considered as a potential rescue therapy for adult patients with SCD who develop life-threatening cardiopulmonary failure. However, most of the existing literature focuses on pediatric populations, and evidence in adult patients remains limited and largely based on case reports and registry data [32,33]. While prior studies have reported aggregate survival rates for SCD patients receiving ECMO, none have differentiated pooled outcomes between VV and VA ECMO. By presenting survival estimates separately for the two modalities, our review provides a novel contribution to the literature, highlighting that VV ECMO is generally associated with more favorable outcomes in ACS-related respiratory failure, whereas VA ECMO is linked to poorer survival, particularly in the context of cardiac arrest or severe hemodynamic collapse.

SCD is a multisystem disorder characterized by chronic hemolytic anemia, endothelial dysfunction, and a hypercoagulable state, which together predispose patients to cardiopulmonary complications such as ACS, pulmonary hypertension, and thromboembolic events [34,35,36]. These underlying pathophysiological mechanisms complicate the initiation of ECMO, which itself carries risks including hemorrhage, thrombosis, and systemic inflammatory responses [37,38,39,40]. Consequently, the decision to deploy ECMO in adult SCD patients should be based on a careful appraisal of disease severity, reversibility, and procedural risk, supported by multidisciplinary clinical judgment. The current evidence base remains limited, underscoring the need for further data to define ECMO’s role and optimize outcomes in this high-risk group.

Given the intrinsic risks of hemolysis, thrombosis, and anticoagulation-related complications in SCD, preventive strategies are essential when considering ECMO. Proactive measures include pre-ECMO optimization with exchange transfusion to reduce HbS levels below 30%, thereby minimizing ongoing sickling and hemolysis. Meticulous anticoagulation management, guided by serial coagulation parameters, is recommended to balance bleeding and thrombotic risks. Where feasible, individualized anticoagulation protocols, such as low-intensity heparin or selective withholding in patients with thrombocytopenia, have shown acceptable safety profiles [41]. Additionally, circuit modifications, including the use of heparin-coated circuits and careful monitoring for plasma-free hemoglobin, may help reduce device-related hemolysis [42]. Early echocardiographic monitoring and integration of adjunctive strategies (e.g., pulmonary vasodilators, prone positioning) further contribute to minimizing morbidity and mortality during ECMO in this high-risk group. Specifically, chronic hemolysis in SCD leads to elevated plasma-free hemoglobin, which may exacerbate ECMO-related hemolysis and increase nitric oxide scavenging, worsening vasoconstriction and pulmonary hypertension. Endothelial dysfunction promotes adhesion of sickled red cells and leukocytes to the vascular wall, amplifying the risk of microvascular occlusion, while the hypercoagulable milieu predisposes to both patient- and circuit-related thrombosis [43]. These interacting mechanisms highlight why SCD patients are uniquely vulnerable to ECMO complications, necessitating tailored preventive and management strategies.

### 4.3. VA ECMO: A High-Risk Intervention with Limited Efficacy

In this systematic review, VA ECMO was primarily utilized in scenarios of severe hemodynamic compromise, such as massive PE, cardiac arrest, and post-cardiotomy shock [44,45]. Clinical outcomes in these cases were generally poor. None of the patients who received VA ECMO for cardiac arrest or PE survived, underscoring the severity of illness and suggesting the limited efficacy of ECMO in these emergent settings. However, one patient who underwent VA ECMO following cardiac surgery did survive, indicating that favorable outcomes may be achievable in carefully selected post-cardiotomy patients.

While the general literature reports more encouraging survival rates with ECMO in patients with PE and cardiac arrest, this trend was not observed in the limited SCD cases identified. For instance, Harwood et al. reported a 61% survival rate among patients undergoing ECMO for PE-related cardiac arrest [46], and Baldetti et al. noted a pooled early mortality of 41.1% in 635 ECMO-supported patients, primarily treated during cardiac arrest [47]. These findings contrast sharply with the SCD cohort, where outcomes appear significantly worse.

Although available data is limited to a small number of case reports, further insight is provided by the Extracorporeal Life Support Organization (ELSO) registry. Among 110 adult SCD patients who received ECMO between 1998 and 2022, overall survival was 52.7% [33]. Notably, among the 50 patients supported with VA ECMO, survival dropped to 38%. In the subgroup requiring extracorporeal cardiopulmonary resuscitation (ECPR), survival was even more dismal, at just 11%.

Postmortem findings in two non-survivors revealed fat embolism and retroperitoneal hemorrhage, evidence of both ECMO-related complications and the inherent vascular pathology of SCD. These findings emphasize the need for careful candidate selection and individualized planning when considering VA ECMO in this high-risk population.

In comparison with non-SCD populations, survival rates with VA ECMO for cardiac arrest or pulmonary embolism are considerably higher, typically ranging from 40 to 60% in registry-based series [46,47]. The markedly poorer outcomes in SCD suggest that baseline disease severity and unique pathophysiology, rather than ECMO itself, may drive much of the excess mortality. This underscores the importance of contextualizing SCD-specific ECMO outcomes within broader ECMO literature, as it reveals how general survival benchmarks may not directly apply to this subgroup.

In addition to acute hemodynamic collapse, some patients may present with refractory VOC, defined as persistent pain, hypoxemia, or progressive organ dysfunction despite maximal conventional management including opioids, hydration, transfusion, and ventilatory support [48]. While VOC alone has rarely been the sole indication for ECMO initiation, its occurrence in combination with ACS or multi-organ dysfunction appears to contribute to worse outcomes. Future studies should distinguish between refractory VOC and ACS-driven respiratory failure when reporting ECMO outcomes, as these may represent clinically distinct subgroups with differing prognoses.

### 4.4. VV ECMO: Improved Outcomes in Acute Chest Syndrome

VV ECMO was employed exclusively in patients presenting with severe ACS who were refractory to conventional treatment. These individuals typically exhibited hypoxemic respiratory failure, frequently triggered by infection or vaso-occlusive crisis. Outcomes with VV ECMO were markedly better compared to VA ECMO; nearly all patients survived, with the only mortality occurring in a patient who initially received VV ECMO but later required conversion to VA ECMO during ECPR.

This favorable trend is supported by data from the ELSO registry, where the survival rate for SCD patients receiving VV ECMO was 61.2% (30 out of 49), notably higher than the 38% observed among those treated with VA ECMO [33]. However, results from Boissier et al.’s retrospective multicenter study, which included 22 SCD patients supported with ECMO for ACS, were less encouraging with an ICU mortality rate of 73% [49]. Although 45% of patients in their cohort received VA ECMO, no statistically significant difference in survival was found between VV and VA modalities. Instead, non-survivors had a higher disease severity at the time of ECMO initiation, reflected by elevated Vasoactive-Inotrope Scores and a greater number of organ failures, highlighting that illness severity, rather than ECMO type alone, may be a key determinant of outcome.

A common therapeutic measure across the reviewed cases was the routine use of exchange transfusion, even in patients with relatively low baseline HbS levels. This approach is aligned with current guidelines recommending a reduction in HbS below 30% in severe ACS, aiming to enhance oxygenation and minimize ongoing sickling. Exchange transfusion plays a complementary role to ECMO in managing severe ACS or multiorgan failure in SCD [49]. While exchange transfusion alone is often sufficient to reverse hypoxemia and vaso-occlusion in many patients, those failing to improve may require escalation to ECMO. Reported outcomes suggest that patients who receive early exchange transfusion prior to ECMO initiation tend to have more favorable courses compared with those in whom ECMO is employed as a last resort [50]. Thus, exchange transfusion should be considered the first-line escalation therapy, with ECMO reserved for cases refractory to transfusion and supportive care. The comparative balance between these two interventions underscores the need for standardized algorithms to guide escalation, incorporating patient selection, reversibility of organ dysfunction, and anticipated risk-benefit profile.

Despite the well-documented bleeding risks associated with systemic anticoagulation, it was generally well tolerated in this group. In select patients with thrombocytopenia, anticoagulation was withheld without observed thrombotic complications, an encouraging observation. In addition, early-stage research has suggested a potential role for inhaled heparin in managing ACS, though more robust clinical trials are needed to support its widespread use.

Echocardiographic assessments frequently revealed RV dilation and strain, consistent with underlying pulmonary hypertension, a serious and often fatal complication of SCD. These findings support the value of early echocardiography to evaluate cardiopulmonary status and guide supportive strategies, including the use of pulmonary vasodilators [51]. In multiple cases, repeat imaging during VV ECMO revealed significant improvements in RV function, likely attributable to improved oxygenation and acid-base balance achieved through extracorporeal support.

Nonetheless, not all VV ECMO cases were successful. One small case series involving three ACS patients with elevated pulmonary artery pressures at the time of VV ECMO initiation reported no survivors. The authors proposed that VA ECMO might be more suitable for such cases [52]. However, considering the consistently high mortality associated with VA ECMO in SCD, and the more favorable outcomes of VV ECMO in both published literature and registry data, VV ECMO remains the preferred initial modality in most cases of refractory ACS.

Furthermore, adjunctive strategies such as prone positioning were successfully integrated in at least two patients, demonstrating that standard lung-protective measures can be safely and effectively combined with ECMO in this population [52]. The duration of VV ECMO support ranged from 3 to 20 days, with most patients weaned within 10 days.

Taken together, these findings support the role of VV ECMO as a viable and effective short-term bridge to recovery in patients with severe ACS who fail to respond to conventional treatment. The favorable pooled survival rate we observed for VV ECMO compared with VA ECMO highlights a key novel insight of this review: ECMO outcomes in SCD are not uniform but instead vary substantially by modality and indication. This reinforces the need for careful modality selection, with VV ECMO preferred for isolated respiratory failure, and extreme caution warranted when considering VA ECMO for circulatory collapse. Favorable survival outcomes reported in both case-based literature and registry analyses suggest that VV ECMO can provide adequate cardiopulmonary support while allowing time for resolution of the underlying inflammatory and vaso-occlusive pathology characteristic of SCD. Moreover, the complication profile of VV ECMO appears more manageable compared to VA ECMO, particularly in the context of bleeding and thrombotic risks.

Ultimately, the experience with ECMO in adult SCD emphasizes the importance of rigorous case selection, ideally involving a multidisciplinary team including hematology, cardiology, and critical care specialists. Careful integration of exchange transfusion, optimization of anticoagulation strategies, and early recognition of refractory VOC or ACS are key to reducing morbidity and mortality. These principles should guide both clinical practice and the design of future prospective studies to better define ECMO’s role in this complex population.

From a clinical standpoint, practical recommendations include prioritizing exchange transfusion as the initial escalation step in severe ACS, reserving ECMO for cases refractory to optimized transfusion and ventilatory strategies. VV ECMO should be favored for hypoxemic respiratory failure without severe circulatory collapse, while VA ECMO should be considered only in select cases with reversible cardiogenic shock, given its poor outcomes in SCD. Multidisciplinary team involvement, early echocardiography, individualized anticoagulation strategies, and adjunctive measures such as prone positioning and pulmonary vasodilators are critical to minimizing morbidity and mortality. Collectively, these measures may help translate the physiological insights into more favorable outcomes for this uniquely high-risk population.

### 4.5. Strengths, Limitations and Way Forward

This systematic review provides a comprehensive synthesis of the existing literature on ECMO use in SCD, particularly during acute crises, consolidating data from 16 case reports encompassing 23 patients. The study meticulously details patient demographics, laboratory findings, management strategies, and outcomes, offering valuable insights into the challenges and potential benefits of ECMO in this high-risk population. By distinguishing between VV and VA ECMO, the review highlights key differences in survival and complication rates, emphasizing the more favorable outcomes associated with VV-ECMO for ACS and respiratory failure. The inclusion of echocardiographic and CT findings further strengthens the clinical relevance of the data, providing a clearer understanding of cardiopulmonary dynamics in these patients. Additionally, the review critically appraises the quality of included studies using the JBI checklist, ensuring methodological rigor. The comparison with registry data and broader literature contextualizes the findings, reinforcing the need for careful patient selection and multidisciplinary decision-making in ECMO deployment for SCD. However, this review is largely descriptive, as the available evidence consisted mainly of single case reports, small case series, and registry data with marked variability in patient characteristics, ECMO indications, management protocols, and outcome definitions. This heterogeneity precluded a formal meta-analysis and limited the ability to generate pooled effect estimates. Also, the overall sample size of this review was very small (23 patients), and the observed differences in outcomes between VV and VA ECMO may be confounded by baseline illness severity, as patients requiring VA ECMO more frequently presented with cardiac arrest or profound hemodynamic compromise. One key methodological limitation of this review is that the included studies were exclusively case reports and small case series, which are inherently prone to reporting bias and lack standardized outcome assessment. Although the JBI checklist was applied to systematically appraise their quality, the absence of randomized or controlled studies limits the strength of causal inference and generalizability of the findings.

To draw more definitive conclusions, a larger number of reported cases is needed, with comprehensive documentation of ECMO management strategies, particularly regarding anticoagulation protocols and associated complications. Although the outcomes with VV ECMO appear favorable, the published literature is limited and likely subject to reporting bias, as unsuccessful VV ECMO cases may be underreported. This limitation hinders a full understanding of the true efficacy and risk profile of VV ECMO in this population.

For clinicians, this review underscores the importance of early recognition of severe cardiopulmonary complications in SCD and the potential role of VV-ECMO as a rescue therapy for refractory respiratory failure, particularly in ACS. However, the high mortality associated with VA-ECMO suggests cautious patient selection, especially in cases of hemodynamic collapse or thromboembolic events. Standardized protocols for anticoagulation, exchange transfusion, and monitoring of right ventricular function should be developed to mitigate risks. For researchers, the limited and heterogeneous nature of current evidence calls for larger, prospective studies to better define ECMO’s efficacy, optimal management strategies, and long-term outcomes in SCD. Collaborative registries, such as ELSO, could be leveraged to pool data and identify predictors of survival. Further investigation into adjunctive therapies, such as pulmonary vasodilators and alternative anticoagulation approaches, is also warranted. Additionally, ethical considerations surrounding ECMO use in advanced SCD with multi-organ dysfunction should be explored to guide clinical decision-making and resource allocation.

## 5. Conclusions

This systematic review highlights the evolving role of ECMO in managing life-threatening cardiopulmonary complications in SCD, particularly in the context of ACS and respiratory failure. While VV-ECMO demonstrates promising survival outcomes, VA-ECMO remains high-risk, with poor efficacy in hemodynamic collapse or cardiac arrest. The findings emphasize the need for individualized patient selection, multidisciplinary collaboration, and careful management of ECMO-related complications. Despite the encouraging results, the current evidence is limited by small sample sizes and potential reporting bias, underscoring the necessity for further research to refine clinical guidelines and optimize therapeutic strategies. Ultimately, ECMO represents a viable but complex intervention in SCD, requiring judicious application and ongoing evaluation to improve outcomes in this vulnerable population. Although our primary focus was on SCD, a small subset of reports involved patients with SCD/beta-thalassemia. The presence of this genotype highlights the broader relevance of ECMO in severe hemoglobinopathies with similar cardiopulmonary complications but also underscores the need for future studies to differentiate outcomes between SCD and beta-thalassemia subgroups more clearly. Future research should prioritize prospective multicenter registries, standardized reporting of ECMO use in SCD, and comparative studies evaluating ECMO against alternative rescue strategies such as early exchange transfusion. Additionally, translational studies exploring the mechanistic interplay between hemolysis, hypercoagulability, and circuit-related complications may inform novel preventive strategies. From a guideline perspective, incorporation of ECMO into SCD-specific critical care pathways, with emphasis on early referral to specialized centers, preferential use of VV ECMO for severe ACS, and stringent criteria for VA ECMO deployment, could provide more structured decision-making and improve patient outcomes.

## Figures and Tables

**Table 1:** Key demographic characteristics of included case reports and series.

Author	Country	Case	Age (Years)	Gender	Concomitant Diseases	Initial Diagnosis
Grazioli et al. [16]	USA	1	38	F	None	VOC, Pulmonary Embolism
	USA	2	48	M	Beta thalassemia	VOC
	USA	3	60	F	Beta Thalassemia and ESRD.	Fulminant colitis requiring colectomy
de Roux et al. [17]	France	4	35	F	CKD; RHD; Aortic and Mitral Valve replacement	Cardiogenic shock, complicated with anuria and acute respiratory failure.
Alashkara et al. [18]	Germany	5	18	F	None	VOC, ACS
	Germany	6	20	F	Beta thalassemia, cholecystectomy, splenectomy, opioid-induced paralytic ileus, left lower DVT	VOC
	Germany	7	38	F	left artificial hip joint replacement, previous pneumonia, and *Escherichia coli*-associated urosepsis	VOC
Belveyre et al. [19]	France	8	19	F	chronic pulmonary hypertension (with no right heart dysfunction) and mitral annuloplasty for the treatment of rheumatic valvopathy. Transfusion iron overload.	VOC and UTI
	France	9	20	F		VOC
Parhar et al. [20]	UK	10	18	F	Exchange for stroke	AGE *Salmonella septicemia*
Grotberg et al. [21]	USA	11	25	M	None	CAP
Sewaralthahab et al. [22]	USA	12	25	F	Hip AVN, PE, iron overload	ARF
Chambers et al. [23]	USA	13	20	F	Pregnant G1P0 25 WKs	CAP/ACS
Tanenbaum et al. [24]	USA	14	32	F	None	ACS
Al-Sawaf et al. [25]	Germany	15	21	M	H/O *Salmonella sepsis* with multiple organ failure, intracranial bleedings requiring surgical evacuation and bone infarction of hips and vertebral bodies	VOC
Gillett et al. [26]	UK	16	20	F	None	VOC
Ciociolo et al. [27]	USA	17	21	F	None	VOC
Offer et al. [28]	Israel	18	31	M	beta-thalassemia, G6PD deficiency, chronic leukocytosis and thrombocytosis	ACS
Yalamarti et al. [29]	USA	19	24	M	None	MRSA Bacteremia
	USA	20	22	F	Lupus nephritis, recurrent ACS, cardiomegaly	ARF
	USA	21	32	M	Nephrogenic DI	ACS
Avgeridou et al. [30]	Germany	22	19	M	None	PE
Hoffmann et al. [31]	France	23	28	F	None	ACS

ESRD: End-stage renal disease; CKD: chronic kidney disease; RHD: Rheumatic heart disease; VOC: Vaso-occlusive crisis; ACS: Acute coronary syndrome; UTI: Urinary tract infection; AGE: Acute gastroenteritis; CAP: Community-acquired pneumonia; AVN: Avascular necrosis; MRSA: Methicillin-resistant *Staphylococcus aureus*; DI: Diabetes insipidus; PE: Pulmonary embolism.

**Table 2:** Key investigations reported in case reports/series.

Author	Case	Initial Echo Findings	CT Chest Findings	HbS%	Other Labs
Grazioli et al. [16]	1	Severe RV dilation	Diffuse GGO and bilateral subsegmental pulmonary embolisms	70%	Hb 6.5 g/dL; Lactate > 17 mmol/L; HCO_3_^−^ 10 mmol; Creatinine 1.9 mg/dL
	2	Severe RV dilation with akinesia of the mid-free wall and normal apical motion. The LV was underfilled with normal but hyperdynamic function.	Bilateral infiltrate on CXR	Not mentioned	Hb 5.7 mg/dL; platelet count 31$\;{\times \;10}^{9}$/L
	3	Severely dilated RV and severely reduced RV systolic function decreased LV size and filling with normal wall thickness and EF 65–70%	Bilateral upper lobe subsegmental pulmonary emboli and bilateral lower lobe consolidation consistent with pneumonia	Not mentioned	Lactate 5–7 mmol/L; Total bilirubin 8.3 mg/dL
de Roux et al. [17]	4	Left ventricular ejection fraction of 0.40, pulmonary systolic arterial pressure of 75 mm Hg, aortic bioprosthetic valve degeneration (mean gradient 68 mm Hg, valve area 0.4 cm^2^), mitral bioprosthetic valve stenosis (mean gradient 9 mm Hg, valve area 1.3 cm^2^), and moderate tricuspid regurgitation.	none	Not mentioned	none
Alashkara et al. [18]	5	Normal PCWP	Bilateral pulmonary consolidations indicating ACS	<30.0% after Ex	WBC 34.3 ${\times \;10}^{9}$/L; Hb 7.8 g/dL; Platelets 157$\;\times \;10^{9}$/L; LDH 1067 U/L; Total serum bilirubin 1.6 mg/dL; Serum creatinine 0.46 mg/dL; Creatine kinase 59 U/L; Troponin I 224 ng/L; CRP 12 mg/dL.
	6	Normal (EF) of 60.0% mild tricuspid valve insufficiency	Bilateral pulmonary consolidations with a positive air-bronchogram in part with ground-glass components	Not mentioned	Hb 4.1 g/dL; WBC 27.3 ${\times \;10}^{9}$/L; Platelets 139.0 ${\times \;10}^{9}$/L; LDH 944.0 U/L; Total serum bilirubin 2.8 mg/dL; Serum creatinine 1.39 mg/dL; CK 117 U/L; Troponin I 5321.0 ng/L; CRP 1.5 mg/dL; Procalcitonin 7.17 ng/mL
	7	Right ventricular strain with dilation of the right ventricle and severe tricuspid regurgitation	Severe bilateral pulmonary infiltrates in addition to a right segmental pulmonary embolism	<5.0% after Exchange	WBC 9.1 ${\times \;10}^{9}$/L; Hb 6.4 g/dL; Platelet count 98.0 × 10^9^/L; LDH 2467 U/L; Total serum bilirubin 2.9 mg/dL; Serum creatinine 0.52 mg/dL; CK 170 U/L; Troponin I 998 ng/L; CRP 22.4 mg/dL; Procalcitonin 1.42 ng/mL
Belveyre et al. [19]	8	Acute cor pulmonale and right ventricular failure	Not mentioned	17% and changed to >5% after exchange therapy	Not mentioned
	9	Acute cor pulmonale with increased SPAP > 60 mmHg, paradoxical septum, mild right ventricular dysfunction (TAPSE at 10 mm), while cardiac output was increased along with moderate mitral regurgitation	Major and bilateral alveolar condensation, cardiomegaly and moderate pleural effusion	31%	Urea 0.97 g/L—Creatinine 18.3 mg/L
Parhar et al. [20]	10	Not mentioned	bilateral lung consolidation ACS	32%	Hb 6.3 g/dL; WBC 37 ${\times \;10}^{9}$/L
Grotberg et al. [21]	11	RV dilation (5 cm) with preserved systolic function.	right lower lobe consolidation	77%	Hb 6.3 g/dL; WBC 15.8 ${\times \;10}^{9}$/L; Platelet count 93 ${\times \;10}^{9}$/L
Sewaralthahab et al. [22]	12	Normal biventricular function, ejection fraction of 60.0% and mild pulmonary hypertension.	Widespread consolidations in all five lobes	Not mentioned	Hb 8.4 g/dL; WBC 16.8 ${\times \;10}^{9}$/L; Platelet count 235 ${\times \;10}^{9}$/L; Total bilirubin 1 mg/dL; LDH 284 U/L; Lactate 0.6 mmol/L
Chambers et al. [23]	13	Not mentioned	Bilateral airspace opacifications, worse on the left	Not mentioned	WBC 53.3 ${\times \;10}^{9}$/L-Hb of 8.1 g/dL; Reticulocyte index of 21.8%
Tanenbaum et al. [24]	14	Right heart failure with a severely enlarged right ventricle, right ventricular volume and pressure overload, mild–moderate tricuspid regurgitation, and a moderately dilated right atrium. Left heart function was normal (ejection fraction approximately 60–65%)	Bilateral lung field opacification.	Difficult exchange	WBC 23.4 ${\times \;10}^{9}$/L; Aspartate amino transferase 768 IU/L; Alanine aminotransferase 385 IU/L; Prothrombin time 32.1; INR 3; Troponin O 0.26 ng/mL
Al-Sawaf et al. [25]	15	Severely reduced left ventricular function with an ejection fraction of less than 20%	Bilateral consolidations	96%	Chlamydia pneumoniae
Gillett et al. [26]	16	High pulmonary artery pressure	Bilateral patchy consolidation	35%	Hb 105 g/L-WBC 7.3 ${\times \;10}^{9}$/L; Platelet count 46 ${\times \;10}^{9}$/L
Ciociolo et al. [27]	17	preserved left ventricular ejection fraction, with reduced right ventricular systolic function and right ventricular overload	bilateral pulmonary opacities	45%	Not mentioned
Offer et al. [28]	18	increased pulmonary pressure and right ventricular failure	bilateral infiltrate, no PE	90%	Not mentioned
Yalamarti et al. [29]	19	elevated PASP with LVEF 65–70	Not mentioned	Not mentioned	Not mentioned
	20	elevated PASP 41% with LVEF 45%	Not mentioned	Not mentioned	Not mentioned
	21	elevated PASP 35% with LVEF normal	Not mentioned	Not mentioned	Not mentioned
Avgeridou et al. [30]	22	a massive impairment of right heart function	PE repeated 2 days later, bilateral infiltrate	49%	Platelet count 50 ${\times \;10}^{9}$/L
Hoffmann et al. [31]	23	Not mentioned	Not mentioned	Not mentioned	Not mentioned

GGO: Ground-glass opacifications; CXR: Chest X-ray; RV: Right ventricle; Hb: Hemoglobin; HCO_3_^−^: Bicarbonate; LDH: Lactate dehydrogenase; WBC: White blood corpuscles; CRP: C-reactive protein.

**Table 3:** Summary of management, complications and outcomes with ECMO in the case reports/series.

Author	Initial Treatment	Indication for ECMO	Type of ECMO	Anticoagulation	Echo Post ECMO	Complications	Duration	Outcome	LOS	Others
Grazioli et al. [16]	Antibiotics Blood Transfusion Alteplase	Pulmonary embolism with shock	VA	None	LV EF 20% with good contractility, RV size was normal with severely reduced systolic function	Retroperitoneal and peritoneal hemorrhage, coagulopathy, CRRT, Brain edema and herniation	3 days	death	3 days	Autopsy: -microscopic pulmonary emboli and fat emboli in alveolar capillaries consistent with ACS. -Fat emboli in glomeruli in the kidneys -Widespread hypoxic–ischemic encephalopathy with occasional petechial hemorrhages and distended capillaries in the cerebrum, suggestive of potential cerebral fat embolism
	Pain management Intubation Anticoagulation	suspected PE with shock + 3 CPRs	VA	Yes	Not repeated	AKI, ALF, Brain herniation	3 days	death	3 days	Autopsy: -Diffuse thrombi and fat emboli in lung capillaries consistent with ACS. -Diffuse fat emboli in the kidneys with acute tubular necrosis. -Petechial hemorrhages and diffuse fat emboli in the brain consistent with cerebral fat embolism.
	Intubation-colectomy	Pulmonary Embolism with shock	VA	None	RV decompression and improved function	abdominal compartment syndrome requiring a decompressive laparotomy, bleeding from the inferior border of the pancreas and diffuse raw surface bleeding consistent with coagulopathy	4 days	death	4 days	
de Roux et al. [17]	Initial treatment with diuretics and dobutamine, Vasopressor administration and cautious fluid infusion, Exchange transfusion	Cardiac Surgery—post CPB	VA	Yes	none	post-CPB hemorrhage	17 days	survival	25 days in ICU and 42 in hospital	
Alashkara et al. [18]	Antibiotics Hydration Opioids Nebulized heparin	ACS	VV	unclear	none	None	49 h	survival	5 days	allogeneic PBSCT from her HLA-identical brother.
	Fluids Opioids, Antibiotics, Exchange Tx, Nebulized heparin	ACS	VV	unclear	none	None	251 h, 30 min	survival	Extubated on day 15	
	Hydration Opioids Antibiotics, Exchange Tx	ACS	VV	Yes	none	transfusion-dependent progressive thrombocytopenia with evidence of a bilateral SDH prior to decannulation	98 h	survival	unclear	subsequent cerebral CT scan, SDH regressed and the patient was transferred to the normal unit.
Belveyre et al. [19]	Antibiotics, Hydration, Opioids, Intubation, Inhalational nitric oxide	ACS	VV	unclear	Regression of the acute cor pulmonale at day 1	None	7 days	survival	Extubated day 16	Had two ECMO
		ACS	VV-spontaneous breathing	Yes	increase RV strain treated with diuretics	Hemorrhagic shock occurred within a few hours due to a large femoral hematoma	19 days	survival	30 days	
Parhar et al. [20]	Antibiotics, Hydration, Opioids, Intubation, Exchange Tx, IVIG	ACS	VV	Yes	none	None	10 days	survival	unclear	
Grotberg et al. [21]	Antibiotics, Hydration, Opioids, Intubation, Exchange Tx, Paralysis, Proning, Inhaled epoprostenol.	ARDS	VV	None	RV function improved	worsened hemolysis (plasma-free Hb of 110 mg/dL) improved with reducing the flow	20	survived	43 days	
Sewaralthahab et al. [22]	Antibiotics, Hydration, Opioids, Intubation, Exchange, Paralysis, Proning	ARDS	VV	Yes	None	spontaneous right pneumothorax, bleeding from tracheostomy site, a urinary tract infection, and DVT	20 days	survival	31 days	
Chambers et al. [23]	Antibiotics, hydration, opioids, intubation, Exchange, delivery	ARDS	VV	unclear	None	none	<10 days	survival	12 days	
Tanenbaum et al. [24]	Antibiotics, hydration, opioids, intubation, Exchange,	ACS	VA thenplus VV	Unclear	None	irreversible severe brain damage.	12 days	death	40 days of withdrawal due to poor neurological recovery	CPR following intubation + transfer + another CPR
Al-Sawaf et al. [25]	Antibiotics, hydration, opioids, intubation, Exchange, SLED	ACS	VV	Unclear	None	None	7 days	survival	Extubated 14 days from decannulation	
Gillett et al. [26]	Antibiotics, Hydration, Opioids, Intubation, Exchange Tx Epoprostenol	ACS	VV	Unclear	Improved PAP	Candidemia	10 days	survival	Not mentioned	
Ciociolo et al. [27]	Antibiotics, Hydration, Opioids, Intubation, Exchange Tx	ACS + barotrauma	VV then VA due to high intorpic support	Unclear	None	None	16 days	survival	Extubated 16 days later and discharged home 44 days later	bilateral subcutaneous emphysema and minor pneumomediastinum after second intubation
Offer et al. [28]	Antibiotics, Hydration, Opioids, Intubation, Exchange, Proning	ARDS + Aspiration pneumonitis	VV	Unclear	None	DVT, watershed brain infarct	7 days	survival	1 month	
Yalamarti et al. [29]	none	ARDS	VV	unclear	not reported	CPR	21 days	death		
	none	ARF	VV than VA	unclear	not reported	cardiogenic shock	>48 days	death		
	none	ARF	VV	unclear	not reported	Brain death with severe hypernatremia and nephrogenic DI—refractory hypoxia	6	death		
Avgeridou et al. [30]	Alteplase, Antibiotics	PE with Cardiogenic shock	Awake VA ECMO + Thrombectomy	Heparin then Argatroban	not reported	HIT	6	survival	22 days	
Hoffmann et al. [31]		ACS	VV	Unclear			12	survival	19 days back to primary hospital	

LOS: Length of stay; LVEF: Left ventricular ejection fraction; CRRT: Continuous renal replacement therapy; ACS: Acute coronary syndrome; AKI: Acute kidney injury; ALF: Acute liver failure; RV: Right ventricle; CPB: Cardio-pulmonary bypass; ICU: Intensive care unit; PBSCT: Peripheral blood stem cell transplantation; SDH: Subdural hematoma; DVT: Deep vein thrombosis; CPR: Cardiopulmonary resuscitation; HIT: Heparin-induced thrombocytopenia.

## Data Availability

The data mentioned in this study is available from published case reports as cited in the paper and is also available from the corresponding author and shall be shared upon a reasonable request.
